# Fingerprint analysis of phenolic acid extract of *Salvia miltiorrhiza* by digital reference standard analyzer with one or two reference standards

**DOI:** 10.1186/s13020-020-00408-9

**Published:** 2021-01-11

**Authors:** Qingjun Wang, Xinlan Yu, Lei Sun, Runtao Tian, Huaizhen He, Sicen Wang, Shuangcheng Ma

**Affiliations:** 1 National Institutes for Food and Drug Control, Beijing, China; 2 Fangshan District Market Supervision and Administration, Beijing, China; 3 Xinjiang Institute for Drug Control, Urumqi, China; 4 Xi’an Jiaotong University, School of Medicine, Xi’an, China; 5 Chemmind Technologies Co., Ltd, Beijing, China

**Keywords:** Substitute reference standards, digital reference standard (DRS), Relative retention time (RRT), Linear calibration using two reference substances (LCTRS), Fingerprints

## Abstract

**Background:**

Fingerprint analysis and simultaneous multi-components determination are crucial for the holistic quality control of traditional Chinese medicines (TCMs). Yet, reference standards (RS) are often commercially unavailable and with other shortages, which severely impede the application of these technologies.

**Methods:**

A digital reference standard (DRS) strategy and the corresponding software called DRS analyzer, which supports chromatographic algorithms, spectrum algorithms, and the combination of these algorithms, was developed. The extensive function also enabled the DRS analyzer to recommend the chromatographic column based on big data.

**Results:**

Various quality control methods of fingerprints of 11 compounds in polyphenolic acid extract of *Salvia miltiorrhiza* (*S. miltiorrhiza*) were developed based on DRS analyzer, involving relative retention time (RRT) method, linear calibration using two reference substances (LCTRS) technique, RRT combined with Photon Diode Array (PDA) method, LCTRS combined with PDA method. Additionally, the column database of samples was established. Finally, our data demonstrated that the DRS analyzer could accurately identify 11 compounds of the samples, using only one or two physical RSs.

**Conclusions:**

The DRS strategy is an automated, intelligent, objective, accurate, eco-friendly, universal, sharing, and promising method for overall quality control of TCMs that requires the usage of fewer RSs.

##  Background

Due to good pharmacological activities and excellent curative effects, traditional Chinese medicine (TCMs) is increasingly popular not only in China but also around the world. Therefore, ensuring the efficient and safe use of TCM is an important issue. Given the complex components of TCMs, it is crucial to carry out a holistic quality control methodology, involving fingerprint technology and multi-components determination technology [1–4]. However, these technologies cannot be realized without reference standard (RS), which has brought great pressure to both providers and users. Firstly, the high price of RS led to a significant increase in the cost of TCM analysis. Besides, some TCM compounds are difficult to be extracted, isolated, and purified, while some are unstable and toxic, all of which lead to problems to the supply of RS. Furthermore, due to the low content of these compounds in TCMs, the preparation of the RS requires a large quantity of TCMs and organic solvents, which is not eco-friendly.

The substitute RS method has been developed as a feasible solution for the problems discussed above. Substitute RS is a method for the qualitative or quantitative determination of another one or more compounds to be measured by one or a few physical RS by using several constant eigenvalues and algorithms [5–8]. Qualitative substitute RS methods include relative retention time (RRT) technique [9–12], extractive reference substance (ERS) method [8–11], linear calibration using two reference substances (LCTRS) approaches [13–15] and Photon Diode Array (PDA) spectrum method [16–18]. Quantitative methods include the relative correction factor method [9–12] and the quantitative ERS technique [9–11]. These methods not only promote the application of multi-components determination and fingerprint analysis for quality control of medicines but also have been proven to be more economical and simple [13–25]. However, the substitute RS method used in the holistic quality control of medicines still has some problems. In particular, the qualitative analysis of chromatographic peaks is the critical issue and the most challenging problem of substitute RS method. For this part, the RRT method and ERS method were adopted by the Pharmacopoeia of several countries, such as Chinese Pharmacopoeia, European Pharmacopoeia, etc. Yet, the drawbacks of the RRT method are large retention time (t_R_) deviation and poor column durability. Also, the reference chromatogram provided by only one chromatographic column by the method of ERS leads to the differences between the actual and reference chromatogram due to the various brands or types of columns. Consequently, scholars have studied the selectivity of reversed-phase columns [26], classified the columns [27, 28], and put forward the method of selection system of columns [29, 30] to solve the problem of blind selection of columns. Nonetheless, the problem of a large prediction deviation of the RRT method has not yet been fundamentally solved.

Compared with the RRT method, the LCTRS method could reduce the deviation of t_R_ prediction [13–15]. However, there is still a challenge for improving the prediction accuracy of t_R_, especially under the circumstances of different types of compounds, or with experiments that are conducted by columns with large differences in retention performance, which may even result in the reverse order of peaks [18]. PDA method may solve the problem of large deviation or reversed the order of peaks to some extent. However, it is difficult to effectively share data or objectively evaluate data in different laboratories, due to a lack of uniform PDA data exchange format among different brands of chromatography workstations [16, 17].

To solve these problems, we introduced the concept of the digital reference standard (DRS) in our previous study [31]. In the present study, a strategy for holistic quality control of TCM was proposed by the DRS analyzer using a phenolic acid extract of *Salvia miltiorrhiza* as an example. DRS analyzer is an algorithm software, which was developed to support the chromatographic algorithm methods of RRT and LCTRS, similarity algorithm of PDA spectrum, as well as the combination of different algorithms mentioned above. It is also a multi-dimensional database, which stores all the original data of the HPLC chromatogram and PDA spectrum during the establishment of the method. These data are not only useful for the calculation by software. Still, they are also crucial for searching and comparison of the chromatographic data by users, finally realizing the recommendation of column based on these data and improving the reproducibility and accuracy of the holistic quality control method. Phenolic acid extract of *S. miltiorrhiza* is the extract of Salviae Miltiorrhizae Radix (Danshen in Chinese), a popular TCM. Salviae Miltiorrhizae Radix is also used as a dietary supplement in other Asian countries, as well as in Europe and America. The design, algorithm, application, and characteristics of DRS analyzer were discussed in this study. Also, a series of quality control methods of fingerprint involving 11 compounds of polyphenolic acid extract of *S. miltiorrhiza* were developed based on DRS method.

##  Methods

### Chemicals and reagents

The phenolic acid extract of *S. miltiorrhiza* was obtained from the National Institutes for Food and Drug Control (NIFDC, Beijing, China). RSs of Sodium Danshensu, Salvianolic acid D, and Lithospermic acid were purchased from Shanghai Yuanye Bio-Technology (Shanghai, China). Reference standards of Protocatechuic aldehyde, Caffeic acid, Rosmarinic Acid, Salvianolic acid B, Salvianolic acid H/I, Salvianolic acid E, Salvianolic acid L, and Salvianolic acid Y were obtained from NIFDC (Beijing, China).

Ethanol, which was analytical grade, was purchased from Sinopharm Chemical Reagent (Shanghai, China). Acetonitrile, methanol, phosphoric acid, and formic acid, which were chromatographic grade, were purchased from Fisher Scientific (Pittsburgh, PA, USA). Deionized water was prepared by a Milli-Q system (Millipore, Bedford, USA).

### Instruments and chromatographic conditions

Chromatographic analysis was performed on Agilent 1260 high-performance liquid chromatography with a DAD detector, ChemStation online control, and offline analysis workstation (Agilent, Santa Clara, CA, USA). Twenty-two columns (Table 1) from seven manufacturers were randomly selected. It is recommended to use at least ten columns from three manufacturers for DRS method research.

**Table 1:** Information of columns

Code	Brand	Type	Specification
Col1	Agilent	Zorbax SB C_18_	4.6 × 250 mm, 5 μm
Col2	Agilent	Zorbax RX C_18_	4.6 × 250 mm, 5 μm
Col3	Shimadzu GL	Inertsil ODS-3	4.6 × 250 mm, 5 μm
Col4	Kromasil	Eternity-5 C_18_	4.6 × 250 mm, 5 μm
Col5	Kromasil	100-5 C_18_	4.6 × 250 mm, 5 μm
Col6	phenomenex	Luna C_18_(2)	4.6 × 250 mm, 5 μm
Col7	Shiseido	Capcell Pak C_18_ SG120	4.6 × 250 mm, 5 μm
Col8	Shiseido	Superiorex C_18_	4.6 × 250 mm, 5 μm
Col9	Shiseido	Capcell Pak C_18_ ACR	4.6 × 250 mm, 5 μm
Col10	Shiseido	Spolar C_18_	4.6 × 250 mm, 5 μm
Col11	Thermo	ODS-2 Hypersil C_18_	4.6 × 250 mm, 5 μm
Col12	Thermo	Hypurity C_18_	4.6 × 250 mm, 5 μm
Col13	Waters	Xterra MS C_18_	4.6 × 250 mm, 5 μm
Col14	Waters	Atlantis T3 C_18_	4.6 × 250 mm, 5 μm
Col15	Waters	Sunfire C_18_	4.6 × 150 mm, 5 μm
Col16	Waters	Xselect HSS C_18_	4.6 × 250 mm, 5 μm
Col17	Waters	Symmetry C_18_	4.6 × 250 mm, 5 μm
Col18	Thermo	Hypersil gold	4.6 × 250 mm, 5 μm
Col19	Agilent	Pursuit C_18_	4.6 × 250 mm, 5 μm
Col20	Agilent	Agilent HC-C_18_(2)	4.6 × 250 mm, 5 μm
Col21	Agilent	Agilent TC-C_18_(2)	4.6 × 250 mm, 5 μm
Col22	Agilent	Polaris C_18_	4.6 × 250 mm, 5 μm

Mobile phase A was 0.1% formic acid-water, and mobile phase B was 0.1% formic acid-acetonitrile. The elution procedure was as shown as below: 20–21.5% B for 0–30 min, 21.5–25% B for 30–35 min, 25–40% B for 35–45 min, 40–95% B for 45–50 min, 95 − 90% B for 50–53 min, 90 − 25% B for 53–60 min. The detection wavelength was 288 nm, and the UV-Vis absorption spectra (190–600 nm) were collected. Column temperature: 30 °C. Flow rate: 1 ml/min. Injection volume: 10 µl.

### Preparation of sample and reference standard solution

The solvent used to dissolve and storage the sample was 25% ethanol-water solution, with pH adjusted to 2.0 by formic acid. The phenolic acids were relatively stable under this condition.

Appropriate amounts (above 16 mg) of phenolic acid extract of *S. miltiorrhiza* and 10 ml solution mentioned above were put into a conical flask, shaken and filtered through a 0.22 µm membrane before use.

An appropriate amount of 11 RSs, including sodium Danshensu, protocatechuic aldehyde, caffeic acid, salvianolic acid D, salvianolic acid E, salvianolic acid H/I, rosmarinic acid, lithospermic acid, salvianolic acid B, salvianolic acid L, and salvianolic acid Y were dissolved by the solution mentioned above to obtain the reference standard solution.

### Software development

####  Data format

DRS Analyzer supports the NetCDF (ANDI) data format [32], which is used for the exchanging and reading of chromatography and spectrometry data. The spectrum data from the PDA detector adopts an extended ANDI format [18]. HPLC instrument vendors such as Agilent and Waters have provided support for PDA spectrum exchanging with the extended ANDI format in their chromatographic workstation through macro or software upgrade.

####  Program design

**Fig. 1:**
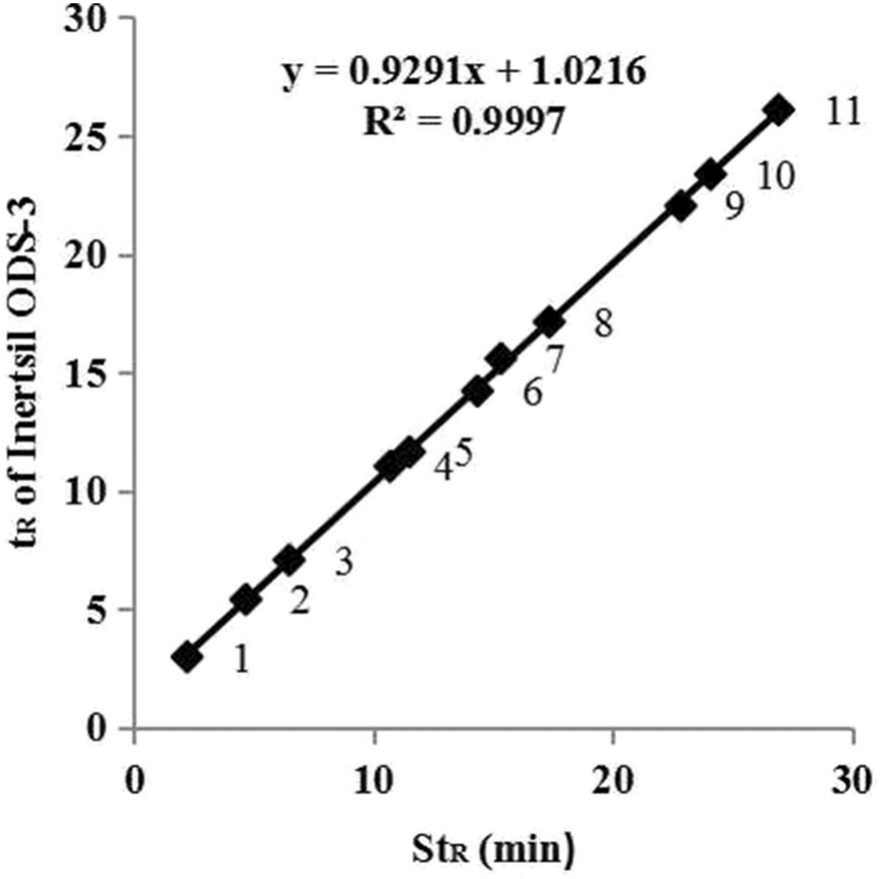
Linear relationship between t_R_ (Inertsil ODS-3) and St_R_. No. 1 to 11 represented Sodium Danshensu, Protocatechuic aldehyde, Caffeic acid, Salvianolic acid D, Salvianolic acid E, Salvianolic acid H/I, Rosmarinic acid, Lithospermic acid, Salvianolic acid B, Salvianolic acid L, and Salvianolic acid Y, respectively

DRS analyzer is developed with C + + language, and Model View Controller (MVC) framework is adopted. It supports the chromatographic algorithm, PDA spectrum algorithm, as well as the combination of different algorithms mentioned above. The chromatographic algorithm includes the RRT method using one RS and the LCTRS method using two RSs. RRT is the ratio between tR of the analyte to the reference compound, which is the reference value for calculating the t_R_ of an analyte. As RRT, St_R_ is also the reference value. But St_R_ is not the ratio; it is the arithmetic average of t_R_ for the same compound on different HPLC systems under the same chromatographic conditions [14]. Also, there is a linear relationship between t_R_ and St_R_ for all compounds [14], as shown in Fig. 1. For the LCTRS method, t_R_ of the two RSs and St_R_ are substituted into linear equation [as expressed in formula (1)] to calculate the t_R_ of the analyte [14]. The similarity algorithm of the PDA spectrum is the cosine method [33].

In addition, the software is a multi-dimensional database, which stores all the original data of the HPLC chromatogram and PDA spectrum during the establishment of the method, and the recommendation of the column could be realized based on these data. The method of recommendation for the column is based on correlation, which is different from the existing recommendation method based on causation [14, 27–30]

1 $${t}_{R}coli=a\times {St}_{R}+b.$$

##  Results

### Optimization of HPLC conditions and method validation

**Fig. 2:**
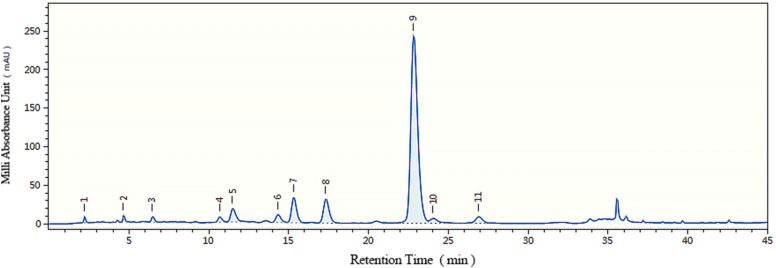
Representative HPLC chromatogram of sample on Column 3 (Inertsil ODS-3). No. 1 to 11 represented the same compounds as Fig.  1

**Fig. 3:**
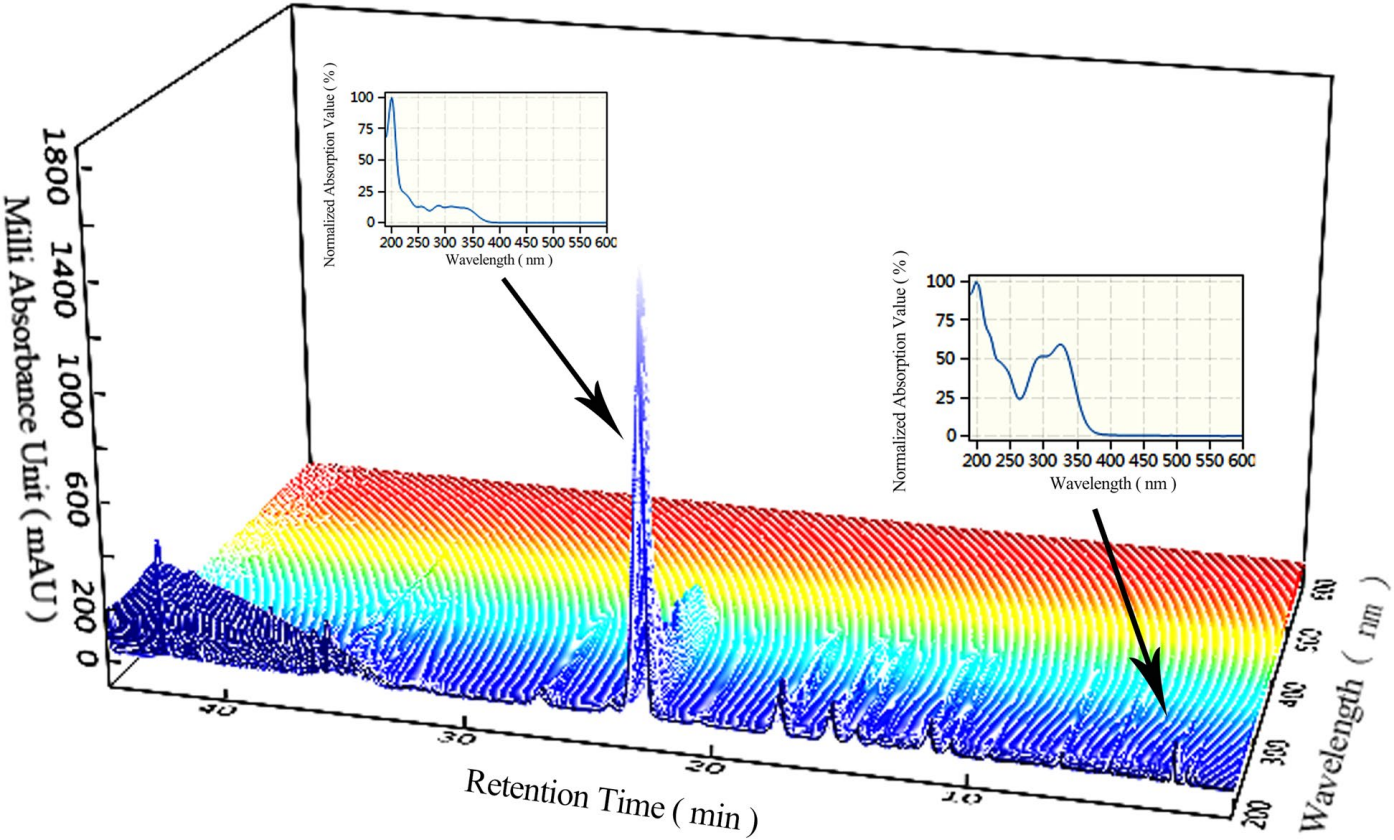
Representative UV-Vis spectra of the sample

The mobile phase was investigated, including the separation effects of methanol and acetonitrile, the differences between phosphoric acid and formic acid, and the influences of column temperature. The gradient elution procedures and flow rates were optimized. The selected chromatographic conditions had good resolution, symmetrical peak shape, and reasonable analysis time. Chromatograms of samples were collected on 22 columns under optimized chromatographic conditions. Representative chromatograms and spectra are shown in Figs. 2, 3. The peaks were identified by the RSs, UV-Vis spectrum and mass spectrum.

Methodological validation experiments were performed on the Agilent Zorbax SB C18 column. The precision (n = 6), stability (12 h, n = 6), and repeatability (n = 6) were tested. The results showed that RSD of the t_R_ of the 11 peaks and the peak areas were both less than 3%, thus meeting the requirements of fingerprint analysis.

### Initialization for the DRS method

**Fig. 4:**
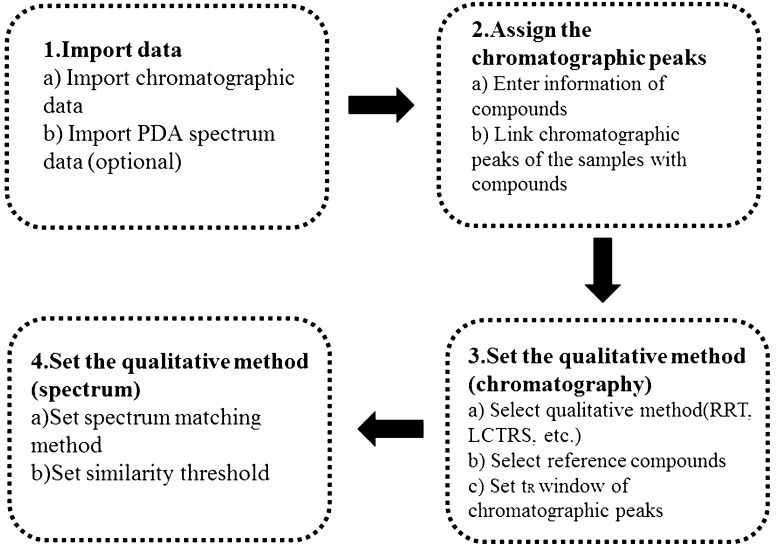
Flow chart of method initialization

**Fig. 5:**
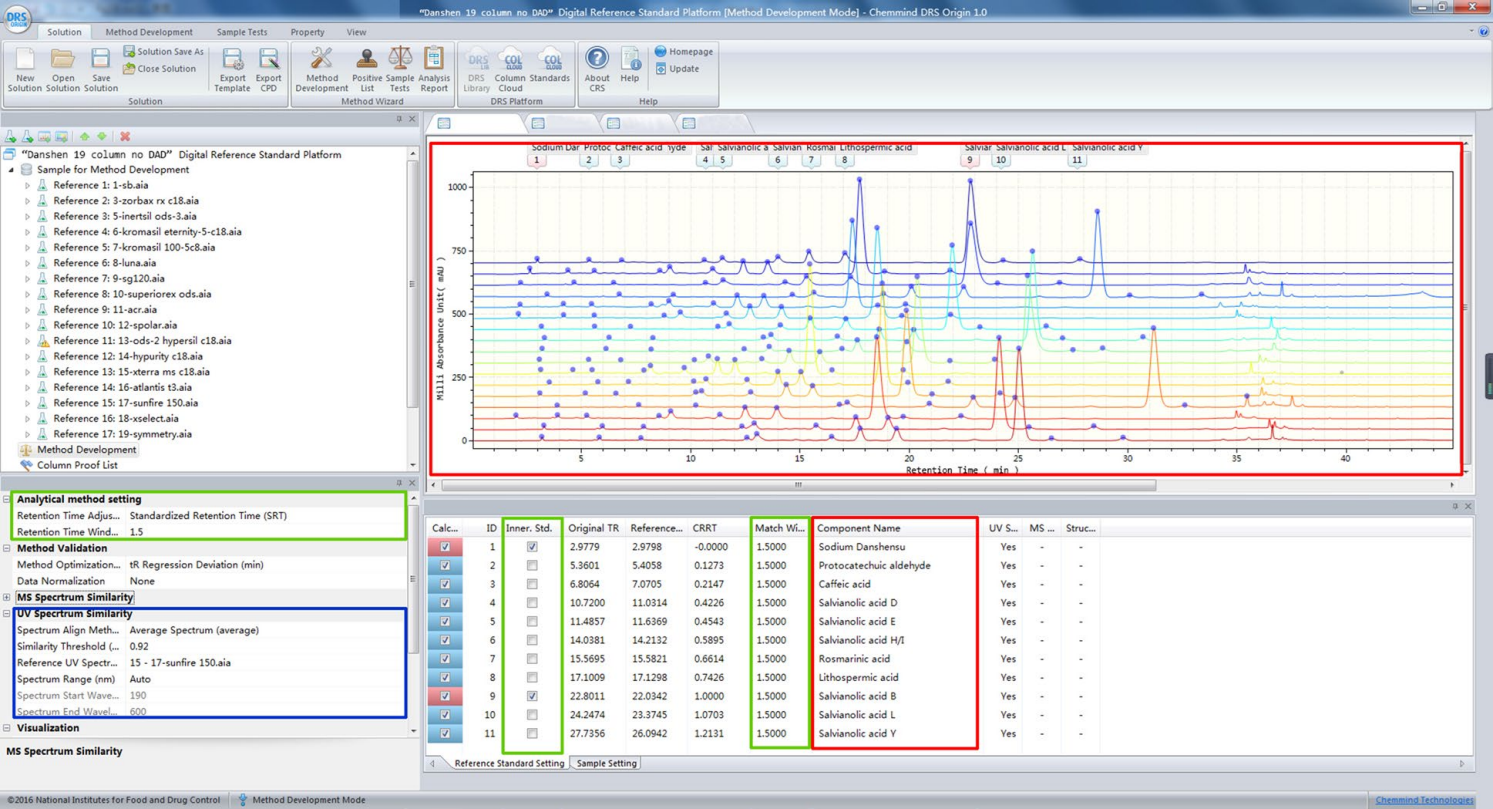
Method initialization on software:  Assign the peaks,  Set the qualitative method (chromatography),  Set the qualitative method (spectrum)

Since the columns of number 1 to 17 could effectively separate 11 peaks of the samples, data on these columns were utilized to initialize the model by steps, as shown in Fig. 4. The first step was data importing. The chromatographic data and corresponding of the samples on columns 1 to 17 were imported into the software, and integration operations such as adding and deleting peaks were performed. The chromatographic data were in ANDI format, with the file name extension “.cdf”. The spectral data were in extended ANDI format, with the file name extension “.nc”. The PDA data was optional. The second step was the peak assignment. Names of the 11 compounds were input into the software, and then the corresponding peaks of the 17 columns and the compounds (the red box part of Fig. 5) were matched one-to-one. The third step was setting the qualitative chromatographic method, taking LCTRS as an example. The t_R_ window of the peak was set to 1 minute. If the t_R_ deviation for the peak was ≤ t_R_ window, the peak could be identified. In this study, peak 1 and peak 9 (recommended to select the peaks close to the first peak and last peak respectively, including the first peak and last peak as well) were selected as two reference compounds, as shown in the green box of Fig. 5. The spectral data were available in the present study, and the fourth step was to establish a spectral qualitative method. As shown in the area of the blue box in Fig. 5, the synthesized spectrum was selected as a spectral matching method, and the similarity threshold was set to 0.95.

### Optimization and evaluation of DRS method

#### Selection of reference compound

Since the selection of the reference compound can significantly affect the accuracy of the RRT and LCTRS method to calculate the t_R_, the optimization was needed. According to our previous studies [14, 34], the general principles for RRT and LCRRS method to select reference compounds were as follows: the t_R_ coverage of the reference compounds was 50–100%, and their non-linear deviation was small enough. The coverage of t_R_ was a reflection of the relative position of reference compound between the first compound and the last compound. For the LCTRS method and RRT method, the calculation of the coverage method was expressed in formula (2, 3), respectively. Since there were various marker compounds in the overall quality control method, even if following the above principle, a large amount of calculation was still required to obtain the optimal reference compounds for the sample under certain chromatographic conditions

2 $$Coverage of {t}_{R}=\frac{{t}_{R2}-{t}_{R1}}{{t}_{Rlast}-{t}_{Rfirst}}.$$

t_R2_ is t_R_ (or St_R_) of second reference compound; t_R1_ is t_R_ (or St_R_) of first reference compound; t_Rlast_ is t_R_ (or St_R_) of last compound; t_Rfirst_ is t_R_ (or St_R_) of first compound [14]

3 $$Coverage of {t}_{R}=\frac{{t}_{Rreference}-{t}_{Rfirst}}{{t}_{Rlast}-{t}_{Rfirst}}.$$

t_Rreference_ is t_R_ of reference compound; t_Rlast_ is t_R_ of the last compound; t_Rfirst_ is t_R_ of the first compound [34].

In the present study, 11 marker compounds and a total of 55 reference compound pairs were obtained, among which about 20 pairs were with t_R_ coverage more than 50%. The software’s method optimization function provided the top 10 reference compound pairs with the highest accuracy, as shown in Table 2. It was revealed that the t_R_ deviation (average deviation of 11 peaks on 17 columns) of the reference compound pair peak 1 and peak 9 was 0.304 min, and the identification rate was 99.5%, ranking 9th. However, the best pair was peak 3 and peak 9, with t_R_ deviation being 0.258 min and identification rate being 99.5%. In comparison, the optimal combination reduced the deviation by 0.046 min.

**Table 2:** Top 10 best reference compound pairs

Reference compound pairs	Peak3 and peak9	Peak2 and peak9	Peak3 and peak8	Peak3 and peak10	Peak2 and peak8	Peak5 and peak9	Peak2 and peak10	Peak5 and peak10	Peak1 and peak9	Peak1 and peak8
t_R_ deviation /min	0.258	0.271	0.274	0.277	0.286	0.292	0.294	0.304	0.304	0.305
Identification rate/%	99.5	99.5	97.3	99.5	97.3	99.5	99.5	99.5	99.5	97.3
The coverage of t_R_/%	64.7	71.9	43.5	70.5	50.7	45.0	77.7	50.8	82.4	61.2

#### Adjustment of t_R_ window

Obviously, on one hand, the smaller the t_R_ window, the more accurate the method was, but on the other hand, the fewer the applicable columns were. The optimal t_R_ window could be determined by the statistical results in the software’s method optimization function. According to Table 3, which showed the average t_R_ deviation on 17 columns of different peaks, the average t_R_ deviation of No.1 to 10 was less than 0.3 min, but for No.11, it was 0.6 min. Therefore, it might be appropriate to set a t_R_ window of 0.8 min to cover the t_R_ deviation of all peaks.

**Table 3:** Average t_R_ deviation of different compounds

Compounds	1	2	3	4	5	6	7	8	9	10	11
t_R_ deviation /min	0.267	0.120	–	0.288	0.173	0.278	0.272	0.184	–	0.148	0.596

**Fig. 6:**
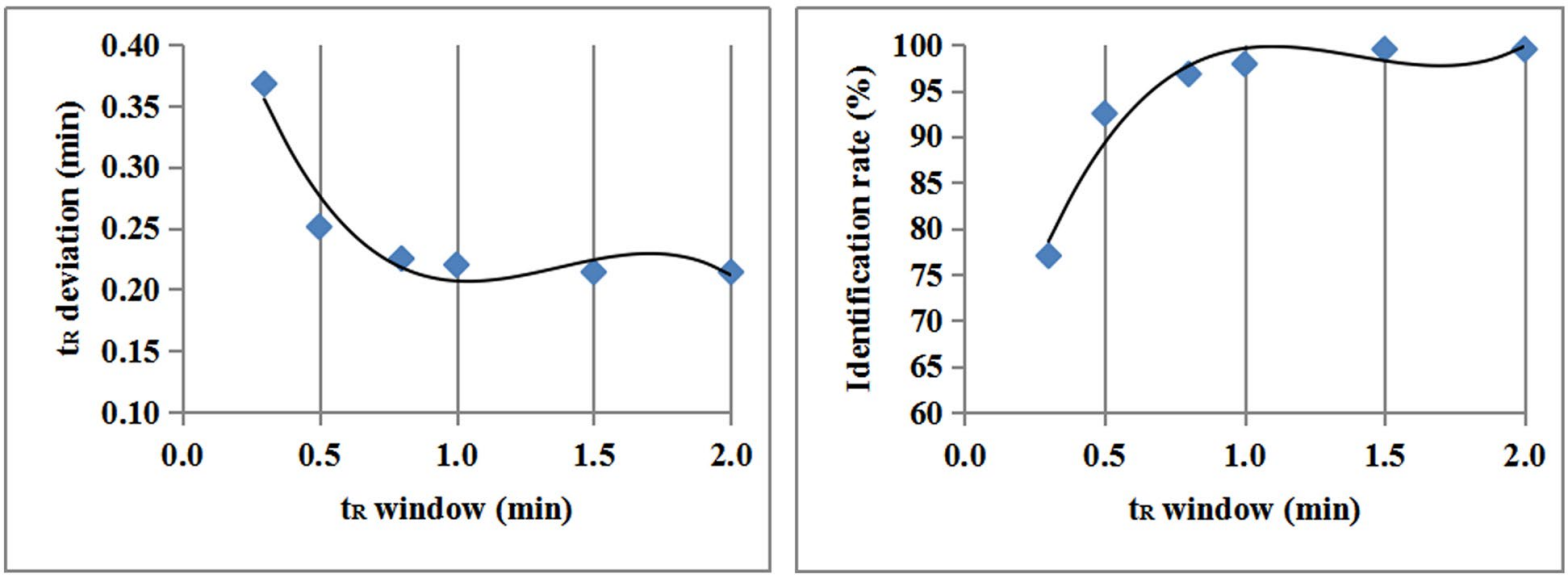
Trend of t_R_ deviation and identification rate with different t_R_ window

To verify this value, different t_R_ windows were set; the t_R_ deviation (average deviation of 11 peaks) and identification rates on different columns are summarized in Table 4; Fig. 6. The obtained results revealed that the windows of 0.3 min and 0.5 min were so narrow that the identification rate was less than 93%, and only a few columns were available, with a proportion less than 53%. Furthermore, the identification rates of 1.5 min and 2.0 min and the available columns were more than 99% and 94%, respectively, and the t_R_ window was considerably large; however, there was a risk of misjudgment. It was demonstrated that 0.8 min and 1.0 min were near the inflection point, being a good balance for both the accuracy and the applicability. Finally, 0.8 min was selected.

**Table 4:** Average t_R_ deviation and identification rate on different columns with different t_R_ window

Code	t_R_ window = 0.3 min		t_R_ window = 0.5 min		t_R_ window = 0.8 min		t_R_ window = 1.0 min		t_R_ window = 1.5 min		t_R_ window = 2.0 min	
	t_**R**_ deviation /min	Identification rate/%	t_**R**_ deviation /min	Identification rate/%	t_**R**_ deviation /min	Identification rate/%	t_**R**_ deviation /min	Identification rate/%	t_**R**_ deviation /min	Identification rate/%	t_**R**_ deviation /min	Identification rate/%
Col1	0.700	45.4	0.350	90.9	0.318	100	0.318	100	0.318	100	0.318	100
Col2	0.400	72.7	0.320	90.9	0.320	90.9	0.320	90.9	0.291	100	0.291	100
Col3	0.147	90.9	0.134	100	0.134	100	0.134	100	0.134	100	0.134	100
Col4	0.297	72.7	0.216	100	0.216	100	0.216	100	0.216	100	0.216	100
Col5	0.257	81.8	0.231	90.9	0.231	90.9	0.210	100	0.210	100	0.210	100
Col6	0.108	100	0.108	100	0.108	100	0.108	100	0.108	100	0.108	100
Col7	0.092	90.9	0.084	100	0.084	100	0.084	100	0.084	100	0.084	100
Col8	0.214	81.8	0.175	100	0.175	100	0.175	100	0.175	100	0.175	100
Col9	0.454	63.6	0.318	90.9	0.318	90.9	0.318	90.9	0.289	100	0.289	100
Col10	0.100	90.9	0.087	100	0.087	100	0.087	100	0.087	100	0.087	100
Col11	1.111	45.4	0.794	63.6	0.617	81.8	0.556	90.9	0.556	90.9	0.556	90.9
Col12	0.175	90.9	0.175	90.9	0.159	100	0.159	100	0.159	100	0.159	100
Col13	0.188	90.9	0.188	90.9	0.170	100	0.170	100	0.170	100	0.170	100
Col14	1.504	27.3	0.645	63.6	0.451	90.9	0.451	90.9	0.410	100	0.410	100
Col15	0.250	81.8	0.204	100	0.204	100	0.204	100	0.204	100	0.204	100
Col16	0.137	90.9	0.125	100	0.125	100	0.125	100	0.125	100	0.125	100
Col17	0.118	90.9	0.108	100	0.108	100	0.108	100	0.108	100	0.108	100
Average	0.368	77.0	0.251	92.5	0.225	96.8	0.220	97.9	0.214	99.5	0.214	99.5

Each peak can be set its own t_R_ window. For example, a window of 0.8 min could be set for peak 11 and 0.5 min for the other peaks. Smaller t_R_ windows were used for the other peaks in this study, which further improved the accuracy of the method and reduced the misjudgment rates.

When the PDA spectrum qualitative function was available, the t_R_ window could be widened. In the current study, it was set to 1.5 min according to the results of Table 4. According to our previous study, t_R_ window was set to 0.5 min [13], 0.6 min, 1.2 min [14], 0.3 min [15] and 0.7 min [18], respectively. Therefore, when only the chromatographic qualitative function was used, the t_R_ window was recommended to be 0.5 to 1.0 min. However, when the PDA spectrum function was obtained as well, it could be widened to 0.5–1.5 min.

#### Comparison of different methods

The software could provide four methods for peak identification, including the RRT method, LCTRS method, RRT combined with the PDA method, and LCTRS combined with the PDA method. The conditions of the four methods optimized according to “3.3.1” and “3.3.2” are shown in Table 5.

**Table 5:** Conditions of different methods

Compounds	1	2	3	4	5	6	7	8	9	10	11
RRT(RRT method, RRT + PDA method )	0.174	0.316	0.423	0.644	0.679	0.830	0.910	1.000a	1.286	1.365	1.523
St_R_/min (LCTRS method, LCTRS + PDA method)	2.980	5.406	7.071^a^	11.030	11.640	14.210	15.580	17.130	22.030a	23.370	26.090

t_R_ windows of RRT method and LCTRS method were both 0.8 min; for RRT combined with PDA method and LCTRS combined with PDA method, t_R_ windows were both 1.5 min, thresholds were both 0.95

^a^reference compound

**Fig. 7:**
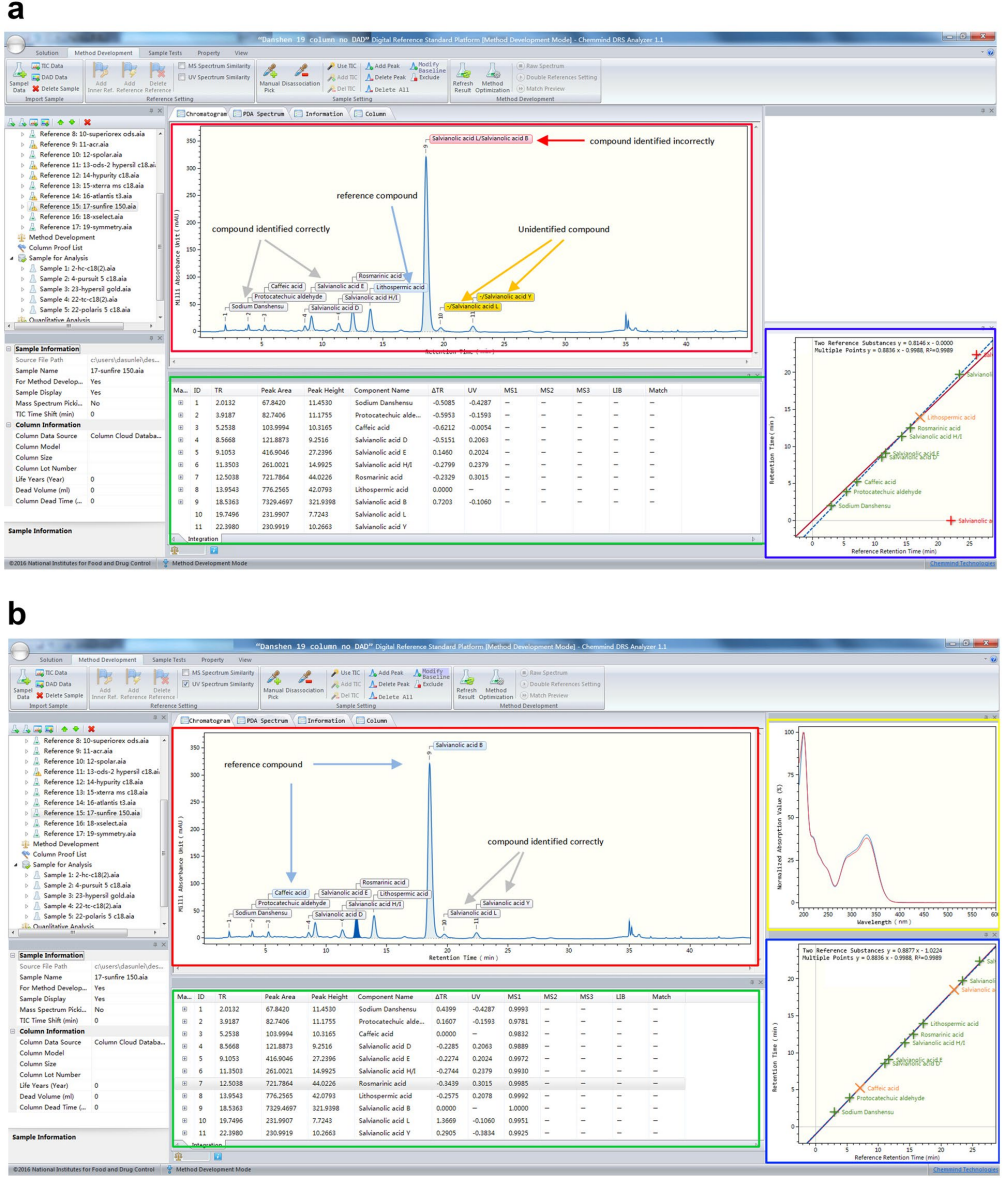
Comparison of RRT method and LCTRS method on column 15 (WatersSunfire, C18).** a** The result of the RRT method,** b** The result of the LCTRS + PDA method ( Qualitative analysis result of peaks,  Information table,  Linear regression result,  Spectrum result)

Taking Col15 (sunfire C18) as an example, Fig. 7a, b showed the results of RRT and LCTRS combined with PDA methods, respectively. The peak identification results in the red box indicated that Salvianolic acid B was incorrectly identified as Salvianolic acid L by the RRT method. Meanwhile, the two peaks of Salvianolic acid L and Salvianolic acid Y could not be identified due to the large t_R_ deviation. Yet, LCTRS combined with the PDA method, accurately identified all peaks. Additionally, the green box revealed the t_R_ deviation of each peak and the similarity of PDA. The blue box provided linear fitting results of t_R_. The yellow box showed the results of the PDA spectrum. The case suggested that LCTRS combined with the PDA method was superior to the RRT method.

The comparison results of t_R_ from column 1 to 17 by the four optimized methods mentioned above are summarized in Table 6. For the number of positive columns (t_R_ deviation ≤ t_R_ window and/or PDA similarity ≥ similarity threshold), it was demonstrated that LCTRS combined with PDA method was the best, with the smallest average t_R_ deviation, the highest identification rate, and the largest amount of available columns. However, LCTRS ranked the highest when only the chromatographic algorithm was used.

**Table 6:** Comparison of different methods (17 columns for method establishment)

Method	Average t_R_ deviation /min	Identification rate/%	Number of positive columns^a^
RRT	0.401	89.8	10
LCTRS	0.225	97.5	12
RRT + PDA	0.343	96.2	12
LCTRS + PDA	0.214	99.5	16

^a^Positive columns were columns on which all peaks could be effectively separated and identified

### Sample tests

**Fig. 8:**
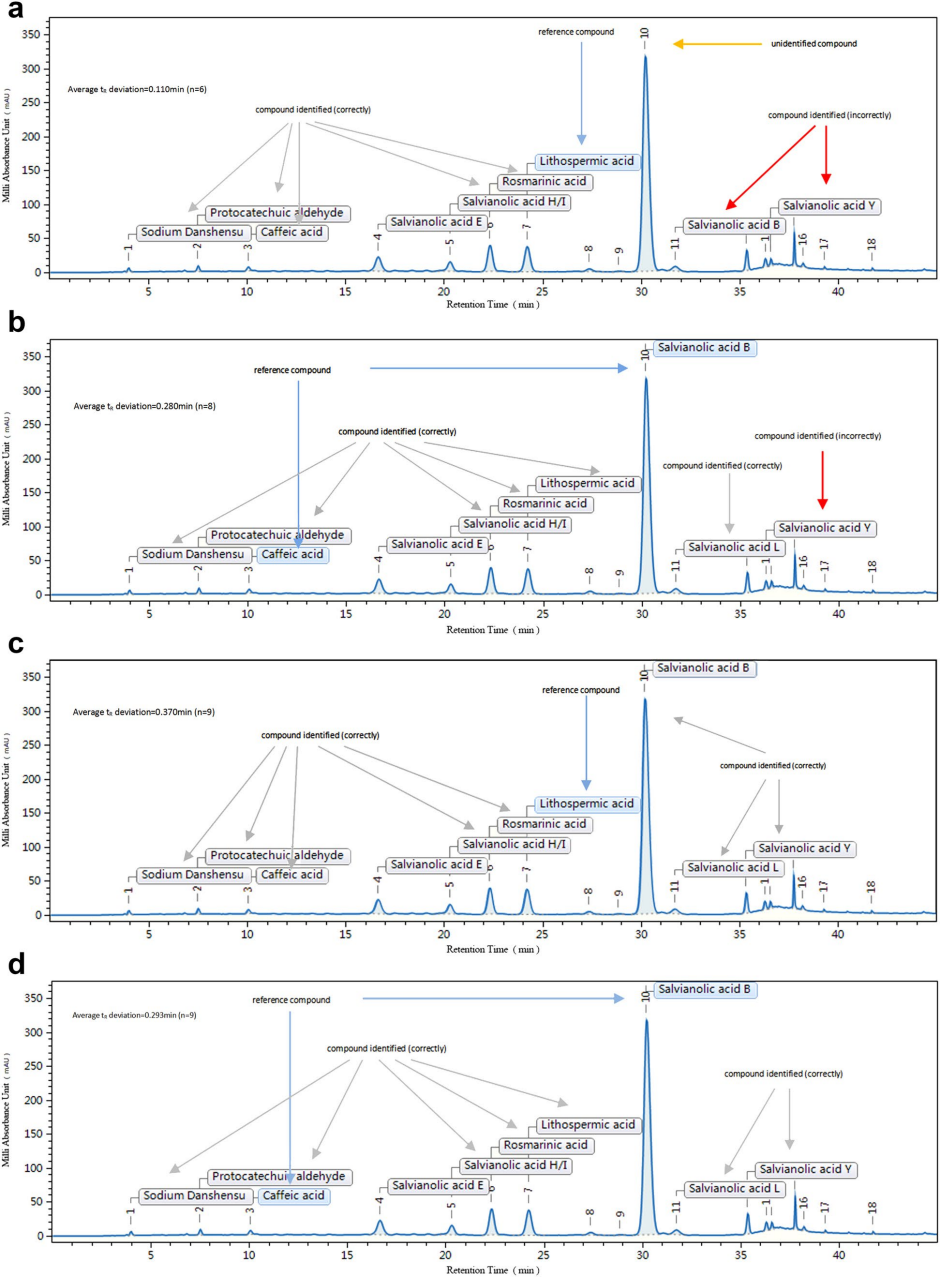
Results of sample tests on column 21 [Agilent TC-C18(2)]. **a** The result of the RRT method, **b** The result of the LCTRS method, **c** The result of the RRT + PDA method, **d** The result of the LCTRS + PDA method

Considering the overlap of Salvianolic acid D peak and Salvianolic acid E peak in the chromatogram on columns 18–22, these columns were used for sample testing rather than method establishment. Three steps were included for sample testing. Firstly, the chromatographic and spectral data were introduced, and the peaks were integrated. Secondly, the reference compounds (peak 3 and peak 9) in the sample chromatogram were assigned. Thirdly, the results were obtained after running the method. The sample test results were exhibited in the same way as shown in Fig. 7, which included the qualitative results of peaks, qualitative result tables, linear fitting results, and spectrum. The peak qualitative results on column Agilent TC-C18 (2) of the four methods are shown in Fig. 8 and A shows the results of the RRT method, which had the smallest t_R_ deviation of 0.110 min. Nevertheless, Salvianolic acid B peak was unidentified; Salvianolic acid L peak and Salvianolic acid Y peak were incorrectly identified. Figure 8b shows the results of the LCTRS method, which had the second smallest t_R_ deviation of 0.280 min. Salvianolic acid L peak was correctly identified, but the Salvianolic acid Y peak was incorrectly identified. The RRT, combined with the PDA method (Fig. 8c) and the LCTRS combined with the PDA method (Fig. 8d) had the same identified results. As shown in figures, the Salvianolic acid L peak and Salvianolic acid Y peak were both correctly identified by the two methods. Still, the LCTRS, combined with the PDA method, had a smaller t_R_ deviation of 0.293 min. Table 7 shows a summary of the comparison results of the four methods established on five columns revealing that the RRT method was still the worst method with the lowest identification rate of 72.7%. On the other hand, LCTRS combined with the PDA method remained the optimal method with a smaller t_R_ deviation of 0.240 min and the highest identification rate of 80.0%.

**Table 7:** Comparison of different methods on five unknown columns, regardless of Salvianolic acid D and Salvianolic acid E

Method	Average t_R_ deviation /min	Identification rate/%
RRT	0.274	72.7
LCTRS	0.185	74.5
RRT + PDA	0.336	80.0
LCTRS + PDA	0.240	80.0

### Column recommendation by database

In the study of the HPLC analysis method, a lot of chromatographic data on different columns are generally collected. However, only the information of column type, such as C18, is provided by the legal standard method. In contrast, data of the brand of the column or related chromatograms are not shown. Nevertheless, these data are indeed valuable, and differences between more useful data (such as with better separation effect, shorter separation time, smaller t_R_ deviation, lower cost of the column) and common data are also meaningful. Therefore, based on the idea of big data, these available data were stored as a part of DRS and used for column recommendation.

Positive and negative columns were defined for column recommendation. Positive columns were referred to columns on which all peaks could be effectively separated and identified. Negative columns were columns on which some peaks could not be separated or identified. In this study, 11 compounds could not be effectively separated on column 21; therefore, this column was considered a negative column for all the four methods (Fig. 8). Column 15 was a positive column for LCTRS combined with the PDA method (Fig. 7b); however, it was negative for the RRT method due to the large retention time deviation of certain compounds (Fig. 7a). For better analysis method reproducibility, future studies should choose the positive column instead of the negative one. For columns that are not on the list of positive or negative columns used, the results, chromatographic data, and PDA spectrum of the column are also meaningful. They can be applied to upgrade and improve the DRS method. Obviously, the positive or negative columns are distinguished for different medicines, different chromatographic conditions, and even for different peak identification methods for the same medicine. The list of the positive and negative columns for the phenolic acid extract of *S. miltiorrhiza* for the four methods is shown in Table 8, while more detailed information is presented on the software database.

**Table 8:** Column recommendations for different methods

Code	Recommendation for RRT method	Recommendation for LCTRS method	Recommendation for RRT combined with PDA method	Recommendation for LCTRS combined with PDA method
Col1	Negative	Positive	Negative	Positive
Col2	Negative	Negative	Negative	Positive
Col3	Positive	Positive	Positive	Positive
Col4	Positive	Positive	Positive	Positive
Col5	Positive	Negative	Positive	Positive
Col6	Positive	Positive	Positive	Positive
Col7	Positive	Positive	Positive	Positive
Col8	Positive	Positive	Positive	Positive
Col9	Negative	Negative	Positive	Positive
Col10	Positive	Positive	Positive	Positive
Col11	Negative	Negative	Negative	Negative
Col12	Negative	Positive	Positive	Positive
Col13	Positive	Positive	Positive	Positive
Col14	Negative	Negative	Negative	Positive
Col15	Negative	Positive	Positive	Positive
Col16	Positive	Positive	Positive	Positive
Col17	Positive	Positive	Positive	Positive
Col18	Negative	Negative	Negative	Negative
Col19	Negative	Negative	Negative	Negative
Col20	Negative	Negative	Negative	Negative
Col21	Negative	Negative	Negative	Negative
Col22	Negative	Negative	Negative	Negative

## Discussion

In the current study, the offline version of the DRS analyzer was used. In order to improve the convenience of data updating and data sharing, an online version should be developed in the future. The future direction of DRS is expected to be with big data, based on which the artificial intelligence could be introduced. In addition, specifications and the guideline of DRS should be studied in the future so as to ensure the authenticity, accuracy, and reliability.

## Conclusions

To the best of our knowledge, the present study is the first that developed a DRS strategy. A series of quality control methods of fingerprints in the phenolic acid extract of *S. miltiorrhiza* was developed based on the DRS analyzer, involving the RRT method, LCTRS method, RRT combined with PDA spectrum method, and LCTRS combined with PDA spectrum method. In addition, the column database of samples was also established. The obtained results revealed the LCTRS combined with the PDA spectrum as an optimal way. The results also demonstrated that DRS analyzer could accurately identify 11 compounds of the samples, using only one or two physical RSs. The strategy significantly reduced the analysis cost and ensured the accuracy and reproducibility of the analysis method.

The DRS strategy adopted in this study has the following advantages. (1) the software automatically processes data, instead of the complex manual calculation, thus saving time and avoiding mistakes in calculation than RRT method and LCTRS method. (2) The results are objective and consistent, avoiding the subjectivity of manual identification than RRT method, ERS method, and LCTRS method. (3) The chromatographic and spectral data formats supported by the software are universal and compatible with mainstream chromatograph workstations; therefore, the popularization and application of the method can be easily realized. (4) It is compatible with a variety of substitute RS methods (such as RRT method, ERS method, and LCTRS method) and supports chromatographic algorithms, spectrum algorithms, and the combination of these algorithms, which has complementary advantages of each method. (5) DRS analyzer is based on the idea of big data to realize the recommendation of the column for different medicines, different chromatographic conditions and different peak identification methods (such as RRT method and LCTRS method) for the same medicine.

In summary, the DRS strategy can effectively reduce the cost of RSs, and achieve higher accuracy and reproducibility than the single substitute RS method. Moreover, it is automated, intelligent, objective, accurate, eco-friendly, universal, sharing, and promising, thus representing a feasible method for overall quality control (such as fingerprint analysis and simultaneous multi-components determination) of TCMs and herbal medicines on different chromatographic columns.

## Data Availability

All data are fully available without restriction.

## Abbreviations

TCMs: Traditional Chinese Medicines
RS: Reference standards
ERS: Extractive reference substance
DRS: Digital reference standard
RRT: Relative retention time
LCTRS: Linear calibration using two reference substances
PDA: Photon diode array
t_R_: Retention time
